# Identification of Immune-Related Gene Signature in Stanford Type A Aortic Dissection

**DOI:** 10.3389/fgene.2022.911750

**Published:** 2022-06-16

**Authors:** Zhaoshui Li, Jumiao Wang, Qiao Yu, Ruxin Shen, Kun Qin, Yu Zhang, Youjin Qiao, Yifan Chi

**Affiliations:** ^1^ Qingdao Medical College, Qingdao University, Qingdao, China; ^2^ Cardiac Surgery Department, Qingdao Hiser Hospital Affiliated to Qingdao University, Qingdao, China; ^3^ Cardiac Surgical Care Unit Department, Qingdao Municipal Hospital Affiliated to Qingdao University, Qingdao, China; ^4^ Hematology Department, Qingdao Hiser Hospital Affiliated to Qingdao University, Qingdao, China; ^5^ Cardiac Surgery Department, Qingdao Municipal Hospital Affiliated to Qingdao University, Qingdao, China

**Keywords:** immune cell infiltration, stanford type A aortic dissection, biomarker, therapy target, bioinformatics

## Abstract

**Background:** Stanford type A aortic dissection (ATAAD) is a common life-threatening event in the aorta. Recently, immune disorder has been linked to the risk factors that cause ATAAD at the molecular level. However, the specific immune-related gene signature during the progression is unclear.

**Methods:** The GSE52093 and GSE98770 datasets related to ATAAD from the Gene Expression Omnibus (GEO) database were acquired. The immune gene expression levels were analyzed by single sample gene set enrichment analysis (ssGSEA). The correlations between gene networks and immune scores were determined by weighted gene correlation network analysis (WGCNA). The different immune subgroups were finally divided by consensus clustering. The differentially expressed genes (DEGs) were identified and subsequent functional enrichment analyses were conducted. The hub genes were identified by protein–protein interaction (PPI) network and functional similarities analyses. The immune cell infiltration proportion was determined by the CIBERSORT algorithm.

**Results:** According to the ssGSEA results, the 13 ATAAD samples from the GEO database were divided into high- and low-immune subgroups according to the ssGSEA, WGCNA, and consensus clustering analysis results. Sixty-eight immune-related DEGs (IRDEGs) between the two subgroups were enriched in inflammatory-immune response biological processes, including leukocyte cell–cell adhesion, mononuclear cell migration, and myeloid leukocyte migration. Among these IRDEGs, 8 genes (*CXCR4*, *LYN*, *CCL19*, *CCL3L3*, *SELL*, *F11R*, *DPP4*, and *VAV3*) were identified as hub genes that represented immune-related signatures in ATAAD after the PPI and functional similarities analyses. The proportions of infiltrating CD8 T cells and M1 macrophages were significantly higher in ATAAD patients in the immune-high group than the immune-low group.

**Conclusion:** Eight immune-related genes were identified as hub genes representing potential biomarkers and therapeutic targets linked to the immune response in ATAAD patients.

## Introduction

Stanford type A Aortic dissection (ATAAD) is one kind of devastating cardiovascular disease (CVD) that accounts for 66% of acute dissections, and it presents an early mortality rate of 1% (Mészáros et al., 2000; De Freitas et al., 2021). Many hypotheses about the causes of ATAAD include the formation of pseudolumen in the aortic medium due to intimal rupture, which is attributed to tearing in the intimal layer of the aorta or bleeding within the aortic wall (Svensson et al., 1999; del Porto et al., 2010; Tchana-Sato et al., 2018). For these patients, early diagnosis and treatment and close follow-up are essential for survival (Pisano et al., 2016). The most valid strategy for ATAAD is immediate surgical repair (Daily et al., 1970; Zhu et al., 2020), which has high technical requirements. However, delivering such care is a challenge for medically underdeveloped areas (Gao et al., 2021). Although immediate surgical intervention has improved patient survival, the mortality rate remains high (Pape et al., 2015). Therefore, the development of novel pharmacologic therapy approaches remains important, which requires a deep understanding of the molecular and cellular bases of ATAAD.

Previous studies showed that blood vessel inflammation caused by autoimmune disease represents a risk factor for damage to the aortic wall that can lead to dissection (Goldfinger et al., 2014; Cifani et al., 2015). Moreover, the proportion of immune cell infiltration is linked to the development of ATAAD (Chen et al., 2021; Gao et al., 2021). For example, the recall and activation of macrophages inside the middle tunic have been identified as the key event in the early phases of ATAAD (Cifani et al., 2015). The monocyte-macrophage system was also reported to play a major role in immune-inflammatory responses in the development of ATAAD (Gao et al., 2021). Besides, immune system-related genes were also found to play key roles in the pathogenesis of ATAAD (Zhang et al., 2022). However, the immune differences that exist among ATAAD patients have not been studied.

The present study aimed to identify the immune cell infiltration-related gene signature that are involved in the development of ATAAD by a bioinformatics approach. This will provide a solid theoretical foundation for further understanding the immune response involved in ATAAD progression.

## Materials and Methods

### GEO Data Acquisition

The raw data about human ATAAD were acquired from GSE52093 and GSE98770 data set (Kimura et al., 2017) of Gene Expression Omnibus (GEO, available at: https://www.ncbi.nlm.nih.gov/geo/) (Barrett et al., 2007) using GEOquery R package (Davis and Meltzer, 2007). The GSE52093 data set contains 12 samples in total, including 5 control samples and 7 disease samples, with the data platform of GPL10558. The GSE98770 data set contains 11 samples in total, including 5 control samples and 6 disease samples, with the data platform of GPL14550. All samples were included in this study. The expression profile was normalized and standardized using limma R package (Ritchie et al., 2015).

### Immune Score Analysis of Samples

Single sample gene set enrichment analysis (ssGSEA) (Hänzelmann et al., 2013) and ImmPort database (available at: https://immport.niaid.nih.gov) (Bhattacharya et al., 2018) were used for the immune related genes set calculation sample grading, and quantitative analysis of immune gene expression levels of aortic dissecting aneurysm. The immune infiltration enrichment scores of samples with disease in the two data sets was estimated, and the samples were divided into high- and low-immunity subgroups based on the ssGSEA results.

### Identification of DEGs and the Enrichment Analysis

For the differentially expressed genes (DEGs) between the high- and low-immunity subgroups, limma R package (Ritchie et al., 2015) was used to identify the significant genes with the threshold of log_2_FC > 1 and *P*_adj_ < 0.05. Ggplot 2 and pheatmap R package were used to visual the DEGs.

Gene Ontology (GO) (Ashburner et al., 2000) and pathway Kyoto Encyclopedia of Genes and Genomes (KEGG) (Ogata et al., 1999) enrichment, and gene set enrichment analysis (GSEA) analysis of DEGs were performed using ClusterProfiler R package (Yu et al., 2012). “Hallmark Gene sets” in MSigDB database (available at: http://www.gsea-msigdb.org/gsea/msigdb) (Liberzon et al., 2015) was selected as the reference gene set in the GSEA analysis. False discovery Rate (FDR) <0.25 and *p*-value < 0.05 were identified as significant. Tidyverse R package was used to conduct Gene Set Variation Analysis (GSVA) analysis (Ferreira et al., 2021), and C2.cp.all.v7.0.symbols” was selected as the reference gene set.

### Weighted Gene Correlation Network Analysis Analysis

The soft threshold was calculated by pickSoftTreshold function, and 13 was the best soft threshold. Then a scale-free network was constructed according to the soft threshold, topology matrix was constructed and hierarchical clustering was carried out. Taking 100 as the minimum number of genes in the module, the identification gene module was dynamically cut and Eigengenes were calculated. According to Eigengenes, the correlation between modules was constructed and hierarchical clustering was carried out to obtain 9 modules. The correlation between modules and clinical features was analyzed by Pearson correlation analysis.

### Identification of Distinguishing Pattern Based on Immune Related Genes

Consensus clustering is a method for determining the number and membership of possible clusters in a data set (microarray gene expression). The ConsensusClusterPlus R package (Wilkerson and Hayes, 2010) was used to conduct consistent clustering on the combined data set using the intersection genes of the genes with the greatest immune association with WGCNA and the genes with differences between the high- and low- immune groups, in order to better distinguish different immune subtypes of ATAAD. In this process, the number of clusters at 9 was set, repeat 100 times to extract 80% of the total samples, clusterAlg = “km”, distance = “euclidean”.

### Venn Analysis of the Co-Gene

Draw venn diagram (available at: http://bioinformatics.psb.ugent.be/webtools/Venn/) (Jia et al., 2021) was used for the co-gene identification.

### Protein-Protein Interaction Network Construction

STRING database (available at: https://cn.string-db.org/) (Szklarczyk et al., 2019) was used to construct the PPI network of hub genes, and Cytoscape software was used for the visualization. The rank of each gene in the network was calculated by CytoHubba (Chin et al., 2014). And the 18 genes with the highest scores were identified as hub genes.

### Functional Similarities Analysis

GOSemSim R package (Yu, 2020) was used to calculate and analyze the functional correlation between key genes.

### Immune Cell Infiltration Analysis

CIBERSORT algorithm (Newman et al., 2019) was used to analyze the immune infiltration among the tissues in the two data sets, to identify the immune cells that were differentially enriched in the different tissues.

### Statistical Analysis

All data calculations and statistical analysis were performed using R programming (available at: https://www.r-projec t.org/, version 4.0.2) (Sepulveda, 2020). For the comparison of the two groups of continuous variables, the statistical significance of the normally distributed variables was estimated using the independent Student t test, and the differences between the non-normally distributed variables were analyzed using the Mann-Whitney *U* test (i.e., Wilcoxon rank-sum test). All statistical *p*-values were bilateral, and *p* < 0.05 was considered statistically significant.

## Results

### Identification of Immune Infiltration-Related Genes in ATAAD

**FIGURE 1:**
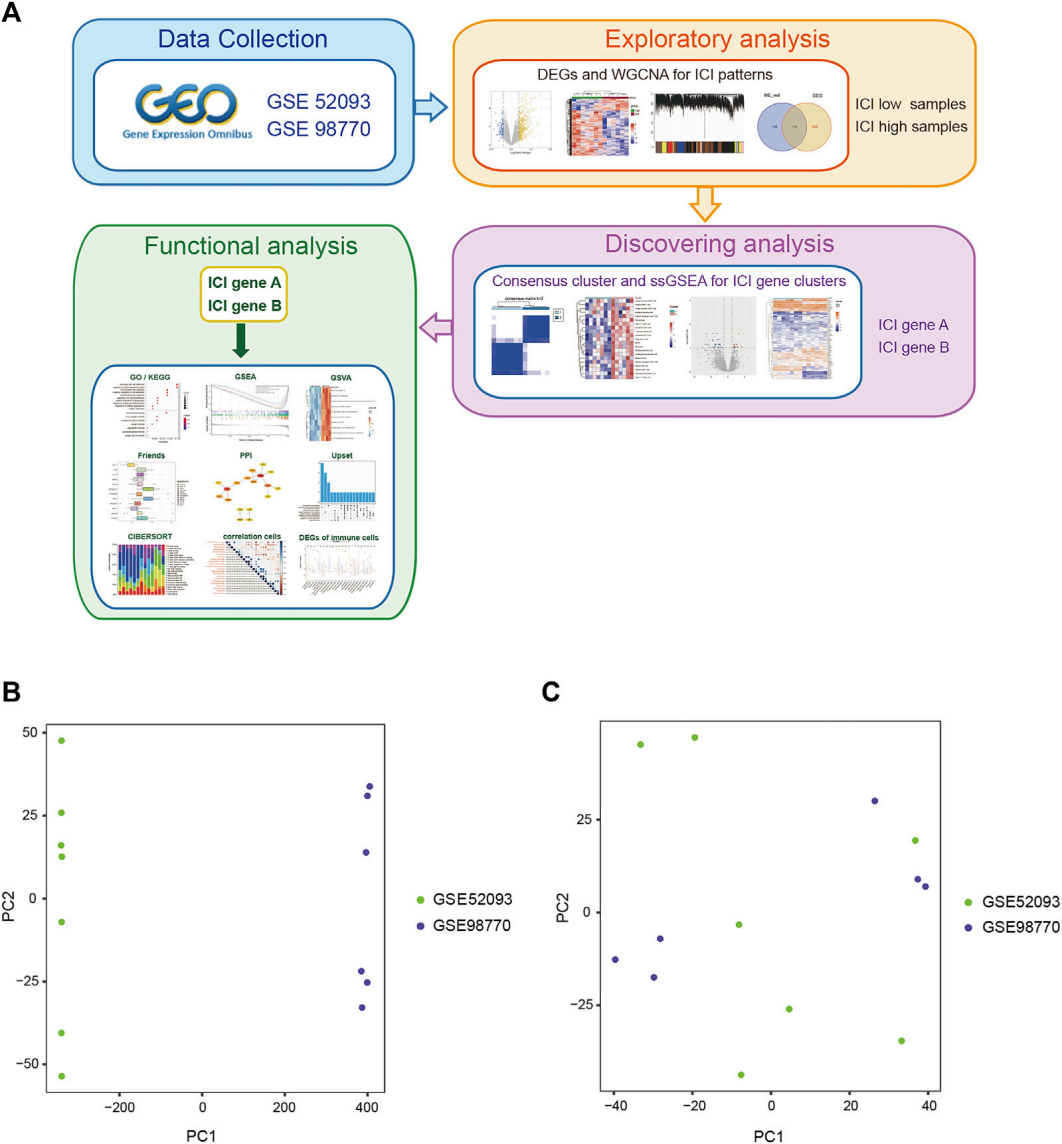
The work flow and PCA analysis of the two GEO data sets. **(A)** The work flow of this study. **(B)** PCA of the two GEO datasets before standardization and batch effect correction. **(C)** PCA of the two GEO datasets after standardization and batch effect correction.

Details on the immune-related cluster construction process and data analyses are shown in Figure 1A. We searched the GEO database, and an RNA expression matrix of the GSE52093 and GSE98770 datasets was acquired and normalized (Table 1, Supplementary Table S1). Principal component analysis (PCA) was performed to show the standardization and batch effect correction of the two datasets (Figures 1B,C).

**TABLE 1:** The information of ATAAD GEO data sets.

Series	Platform	Normal	ATAAD samples	Country	Year	Contributor
GSE52093	GPL10558	5	7	China	2014	Pan S
GSE98770	GPL14550	5	7	Japan	2017	Naoyuki Kimura

**FIGURE 2:**
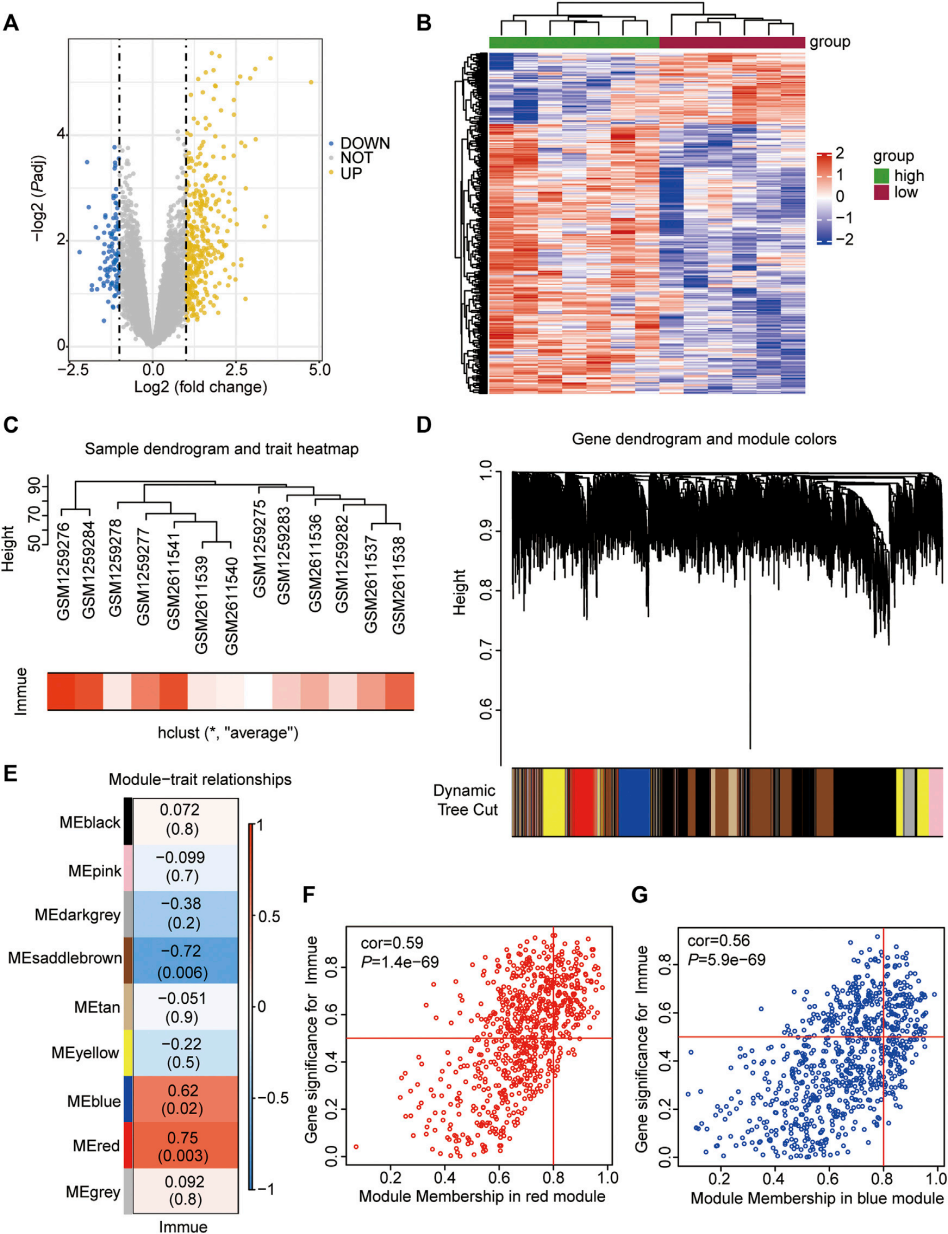
Immune infiltration-related DEGs and WGCNA analyses. **(A)** Volcano Plot showing the DEGs in the high- vs. low-immune subgroups. The yellow dots represent up-regulated genes, the blue dots represent down-regulated genes, and the gray dots represent genes with no significant difference. DEGs were identified with the threshold of adjust *p*-value<0.05 and Fold change>2. **(B)** Heatmap showing the DEGs in the high- vs. low-immune subgroups. The abscissa showed the patient ID, and the ordinate showed genes with different expression. The red represents down-regulated genes, while the blue represents up-regulated genes. **(C)** Sample dendrogram and trait heatmap showing the ATAAD GEO samples clustering. The red represents samples with high immune, the white represents samples with low immune. **(D)** Gene dendrogram and module colors showing the gene clustering. The different color represents different expressional module. **(E)** Heatmap showing the co-relationship of different modules and immunophenotype. **(F)** Scatter plot showing the correlation between immunophenotype and genes in red module. **(G)** Scatter plot showing the correlation between immunophenotype and genes in blue module.

To analyse the effect of immune-related gene expression on ATAAD, ssGSEA was performed, and then the 13 ATAAD samples were divided into high- and low-immunity subgroups according to the ssGSEA results (Supplementary Table S2). DEGs between the two subgroups were identified and were shown in the volcano plot, which indicated that a total of 363 genes were differentially and significantly expressed between the two groups (Figure 2A). Among these genes, 287 were upregulated and 76 were downregulated in the high-immunity group compared to the low-immunity group (Figures 2A,B, Supplementary Table S3).

### Identification of Modules Associated With Immune Scores

WGCNA was performed to identify coexpressed gene modules of the two datasets to explore the relationship between gene networks and immune scores. The soft threshold power was determined based on the scale-free R2 (R2 = 0.95). After merging similar modules, a total of 9 modules were identified and clustering dendrograms were presented (Figures 2C–E). A heatmap was drawn to show correlated modules, which indicated that the red (cor = 0.75, *p* = 0.003) and blue (cor = 0.62, *p* = 0.02) modules were most positively correlated with immunity, whereas the brown (cor = −0.72, *p* = 0.006) module was negatively correlated with immunity (Figures 2E,F). We focused on those genes being positively associated with immunity, therefore, genes colored red (n = 729) and blue (n = 821) were selected for the following analyses (Supplementary Table S4).

### Consistent Unsupervised Cluster Analyses of Immune-Related Gene Clusters

**FIGURE 3:**
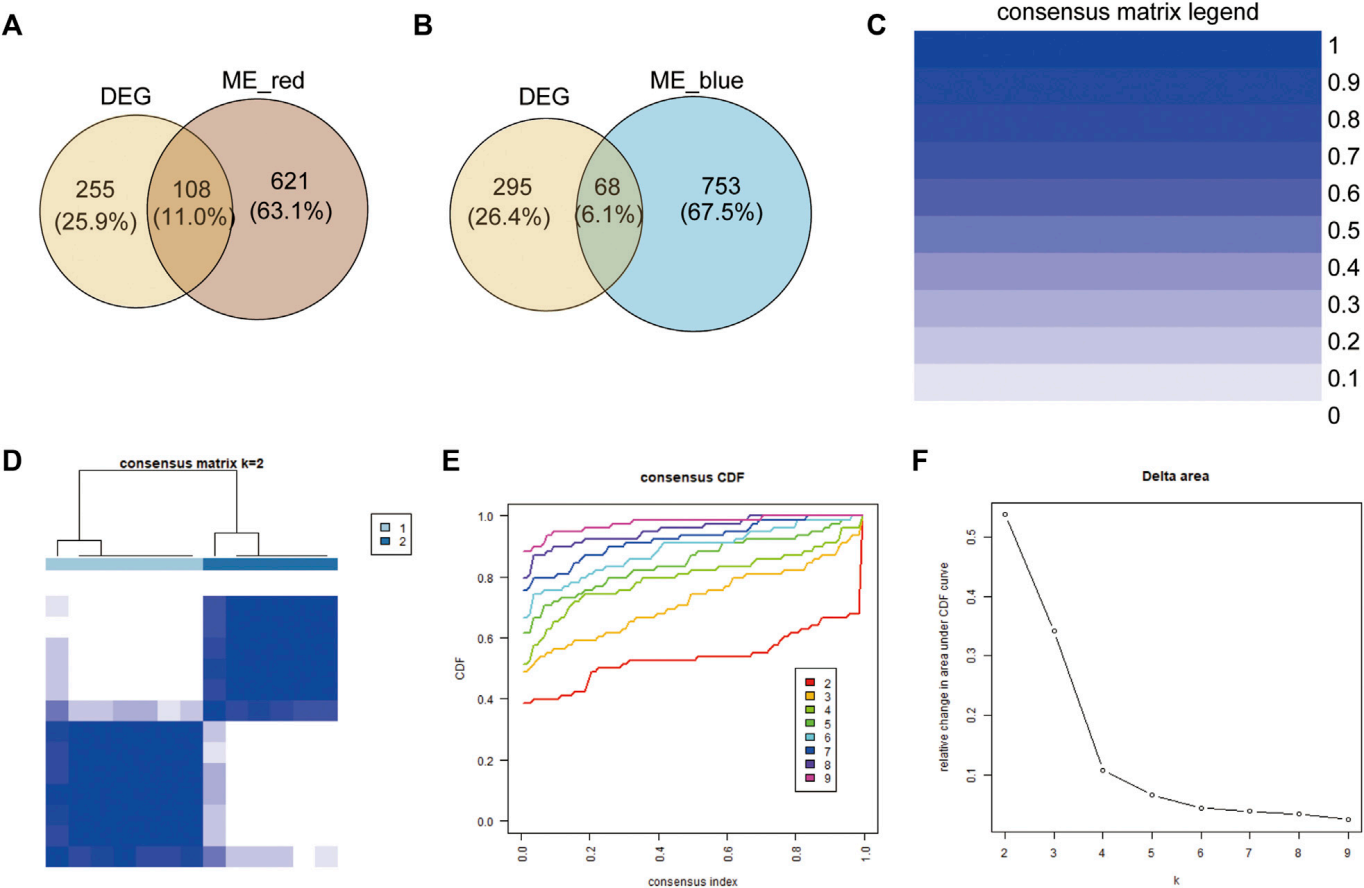
Construction of immune-related gene clusters. **(A)** Venn showing the co-genes between red module genes and DEGs. **(B)** Venn showing the co-genes between blue module genes and DEGs. **(C)** Consistency Unsupervised clustering color legend. **(D)** The consistency matrix of all data sets with k = 2. **(E)** Consistent cumulative distribution showing the cumulative distribution function with different values of k, which is used to judge the optimal value of k. **(F)** Delta area map.

To further clarify the biological differences in immune cell infiltration patterns, the common genes (co-genes) between the red module and DEGs of the two immune subgroups (Figure 3A) and between the blue module and DEGs of the two immune subgroups (Figure 3B) were determined. According to the Venn results, a total of 176 co-genes were used to perform a consistent unsupervised cluster analysis. We found that when k = 2, the delta area of subtype aggregation decreased significantly and entered the plateau (Figures 3C–F). Hence, the 13 patient samples were divided into cluster 1 and 2 [immune-differential gene (IDEG)-related clusters] based on the consistent unsupervised cluster analyses.

### Identification of DEGs Between the Two Immune-Related Clusters

**FIGURE 4:**
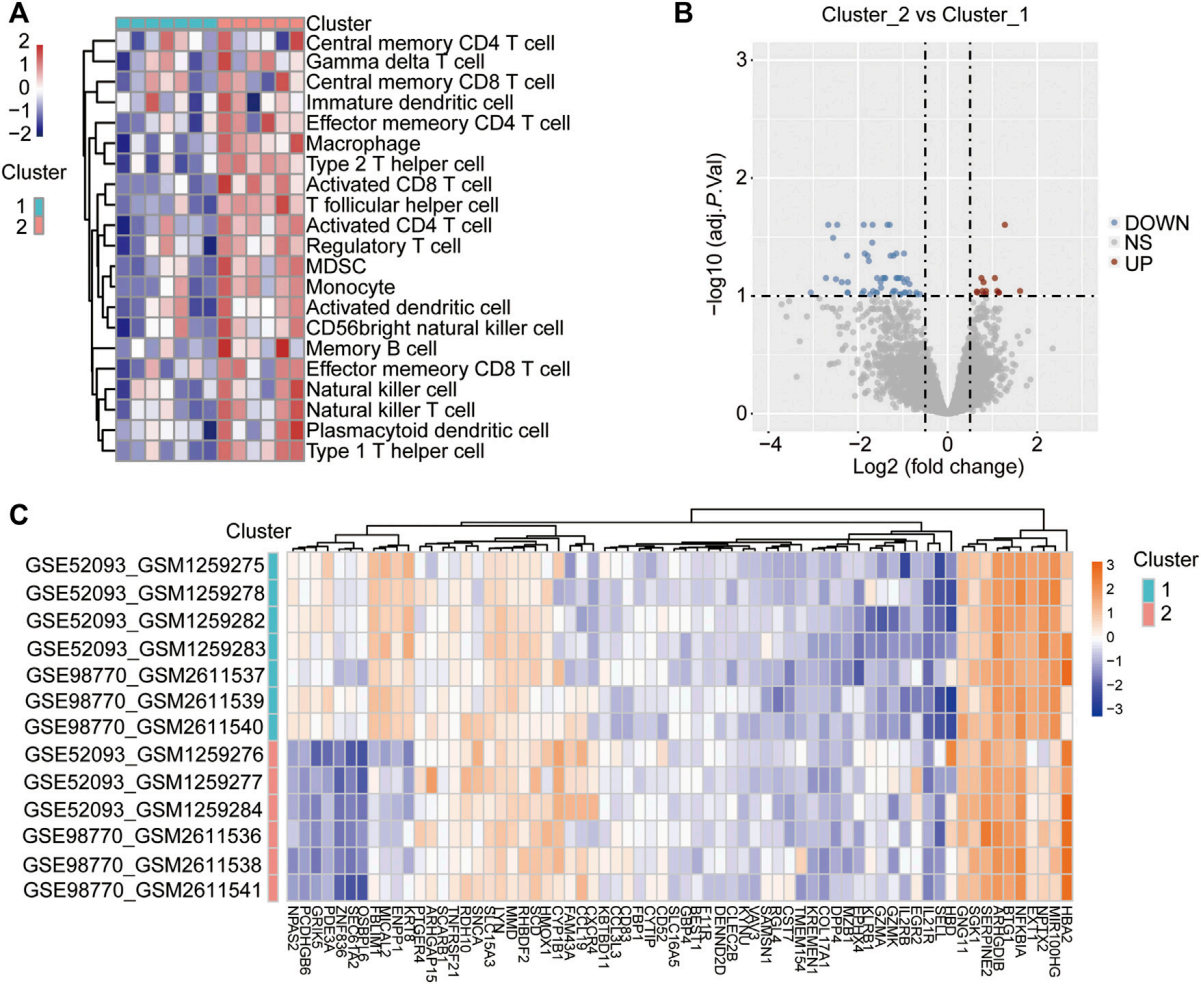
Identification of DEGs between the two immune-related clusters. **(A)** Heatmap shows the difference in immune cell infiltration between the different immune-related clusters via ssGSEA. The red represents activated immune cells and blue represents suppressed immune cells in cluster 2. **(B)** Volcano Plot showing the DEGs in the cluster 2 vs. cluster 1 subgroups. The red dots represent up-regulated genes, the blue dots represent down-regulated genes, and the gray dots represent genes with no significant difference. DEGs were identified with the threshold of adjust *p*-value<0.05 and Fold change>2. **(C)** Heatmap showing the DEGs in the cluster 2 vs. cluster 1 subgroups. The red represents up-regulated genes, while the blue represents down-regulated genes.

ssGSEA was used to analyse the differences in immune cell infiltration between the two different immune-gene subtypes, with the heatmap showing that the infiltration of immune cells was significantly higher in cluster 2 than in cluster 1 (Figure 4A), which indicated that cluster 2 represented a high immune-gene subgroup. Then, the DEGs between the two clusters were identified to further evaluate the biological characteristics. The results showed that a total of 68 genes were differentially expressed between the two immune gene subgroups (Figures 4B,C, Supplementary Table S5), and we named these genes immune-related DEGs (IRDEGs).

### Functional Enrichment Analysis of the IRDEGs

**FIGURE 5:**
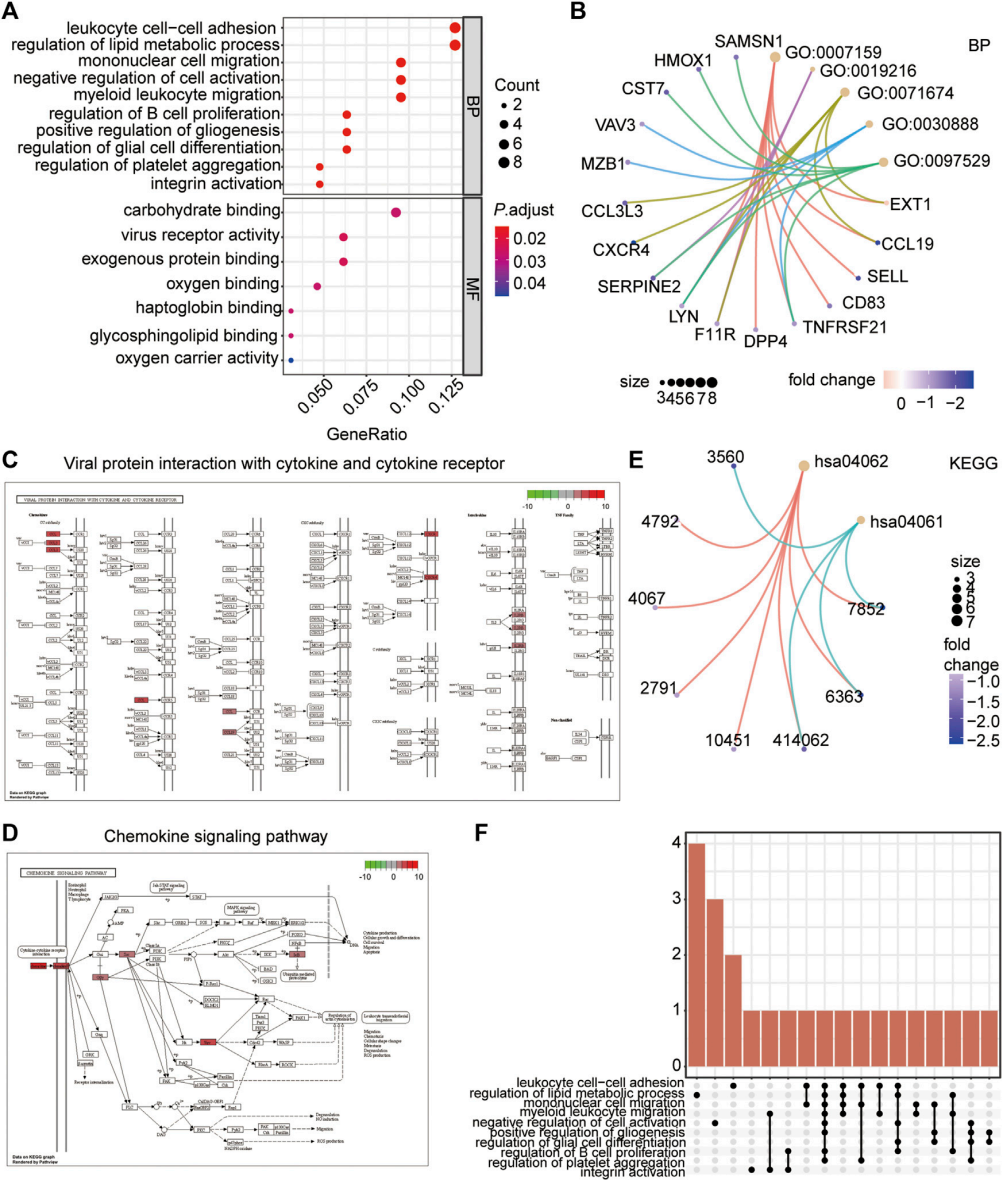
Functional enrichment analysis of the IRDEGs. **(A)** Bubble plot showing the top 10 GO terms (biological processes and molecular function) the IRDEGs enriched in. **(B)** The network diagram showing the correlation of genes with the top 5 biological processes. The node color represents the gene expression level, red node represents the up-regulated genes, and blue represents the down-regulated genes. The size of the circle indicates the correlation. **(C)** The KEGG pathway of Viral protein interaction with cytokine and cytokine receptor. **(D)** The KEGG pathway of Chemokine signaling Pathway. **(E)** The network diagram showing the correlation of genes with the two KEGG pathways. The node color represents the gene expression level, red node represents the up-regulated genes, and blue represents the down-regulated genes. The size of the circle indicates the correlation. **(F)** The Upset diagram showing the intersection genes of different GO terms.

To analyse the biological processes (BP), molecular functions (MF), and KEGG pathways in which the IRDEGs were enriched in, GO and KEGG enrichment analyses were performed (Figures 5A–D). The results showed that ATAAD-related IRDEGs were mainly enriched in BPs of leukocyte cell–cell adhesion, regulation of metabolic processes, mononuclear cell migration, and myeloid leukocyte migration (Figure 5A; Table 2). Among these, leukocyte cell-cell adhesion was the most prominent with the largest number of genes (Figure 5B; Table 2). The enriched MFs of these genes were carbohydrate protein binding, virus receptor activity, and exogenous protein binding (Figure 5A). The KEGG pathway enrichment analysis showed that IRDEGs were enriched in two biological pathways: viral protein interactions with cytokines and cytokine receptors (Figure 5C; Table 3), and the chemokine signaling pathway (Figure 5D; Table 3). The later was more remarkable which was enriched by seven IRDEGs (Figure 5E; Table 3). The correlations among different biological process terms showed that leukocyte cell–cell adhesion and mononuclear cell migration had the largest number of genes that were enriched in other terms (Figure 5F). These results indicated that leukocyte cell–cell adhesion and mononuclear cell migration were the main bioprocesses in which IRDEGs were enriched.

**TABLE 2:** Top 10 GO enrichment of IRDEGs.

Term	ID	Description	*P*.adjust
BP	GO:0007159	leukocyte cell-cell adhesion	0.014482367
BP	GO:0019216	regulation of lipid metabolic process	0.014482367
BP	GO:007167	mononuclear cell migration	0.014482369
BP	GO:005086	negative regulation of cell activation	0.014482366
BP	GO:0097529	myeloid leukocyte migration	0.014482369
BP	GO:003088	regulation of B cell proliferation	0.014482366
BP	GO:001401	positive regulation of gliogenesis	0.0144823669
BP	GO:004568	regulation of glial cell differentiation	0.0144823669
BP	GO:009033	regulation of platelet aggregation	0.0144823669
BP	GO:003362	integrin activation	0.0144823669
MF	GO:0030246	carbohydrate binding	0.02718492
MF	GO:0001618	virus receptor activity	0.02403452
MF	GO:0140272	exogenous protein binding	0.02403452
MF	GO:0019825	oxygen binding	0.02718492
MF	GO:0031720	haptoglobin binding	0.02733267
MF	GO:0043208	glycosphingolipid binding	0.02733267
MF	GO:0005344	oxygen carrier activity	0.04694502

**TABLE 3:** KEGG pathway enrichment of IRDEGs.

Ontology	Description	*P*.adjust
KEGG_PATHWAY	hsa04062: Chemokine signaling pathway	0.005
KEGG_PATHWAY	hsa04061: Viral protein interaction with cytokine and cytokine receptor	0.018

### GSEA and GSVA Enrichment Analysis of the Two Immune-Related Clusters

**FIGURE 6:**
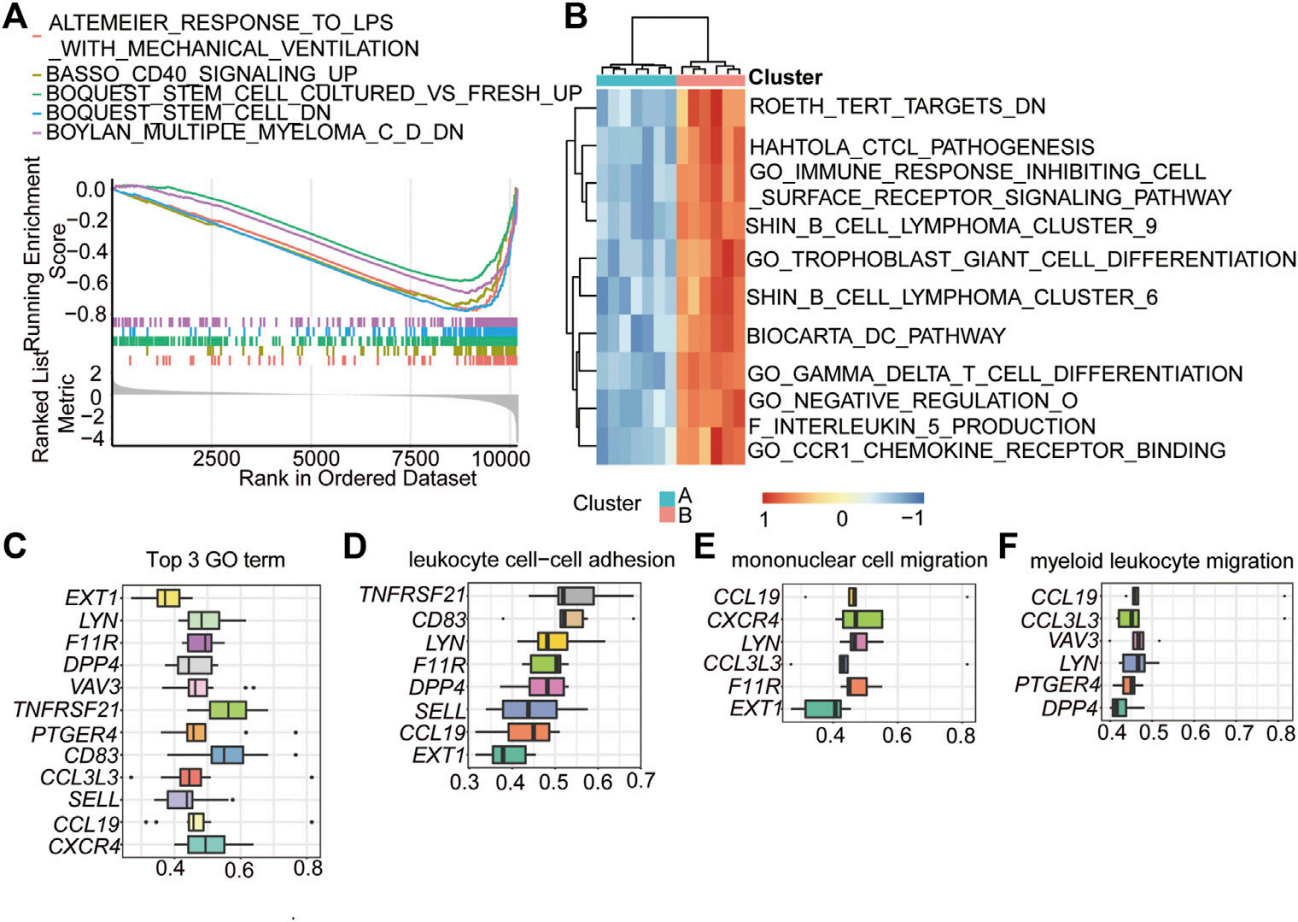
GSEA and GSVA enrichment analysis of the two immune-related clusters. **(A)** GSEA showing the top 5 enriched pathways in HALLMARK set of cluster 2. Each line represents a particular term in a unique color. The up-regulated genes located near the left, while the down-regulated genes located near the right. The threshold was FDR q < 0.06. **(B)** GSVA showing the top 10 pathways enriched in the cluster 2. **(C)** functional similarities analysis of the genes played important roles in the top 3 biological processes. **(D–F)** functional similarities analysis of the genes played important roles in leukocyte cell-cell adhesion **(D)**, mononuclear cell migration **(E)**, and myeloid leukocyte migration **(F)**.

GSEA and GSVA were also performed to analyse the biological pathways enriched in the two immune subtypes. The GSEA results showed that the altemeier response to lps with mechanical ventilation, basso CD40 signalinf up, boquest stem cell cultured vs. fresh up, boquest stem cell dn, and Boylan multiple myeloma C D dn pathways were significantly enriched in the cluster 2 subgroup (Figure 6A; Table 4). The GSVA results showed that immune-related genes mainly influenced following pathways: roeth tert targets dn, hahtola ctcl pathogenesis, immune response inhibiting cell surface receptor signalling pathway, shin b cell lymphoma cluster 9, trophpblast giant cell differentiation, shin b cell lymphoma cluster 6, and biocarta dc pathway (Figure 6B), and these pathways were activated in cluster 2.

**TABLE 4:** GSEA enrichment of immune-related clusters.

ID	setSize	NES	*P*.adjust
ALTEMEIER_RESPONSE_TO_LPS_WITH_MECHANICAL_VENTILATION	82	−2.556893	5.97E-09
BASSO_CD40_SIGNALING_UP	72	−2.429087	5.97E-09
BOQUEST_STEM_CELL_CULTURED_VS._FRESH_UP	316	−2.210047	5.97E-09
BOQUEST_STEM_CELL_DN	135	−2.723678	5.97E-09
BOYLAN_MULTIPLE_MYELOMA_C_D_DN	161	−2.364818	5.97E-09

To further explore the internal mechanisms affecting these biological processes and pathways, a functional similarities analysis based on GO was conducted to determine the key genes (hub-genes) involved in these pathways. The results showed that a total of 12 genes were identified as hub genes in the top 3 GO terms (Figure 6C). Among these genes, C-X-C motif chemokine receptor 4 (*CXCR4*), C-C motif chemokine ligand 19 (*CCL19*), selectin L (*SELL*), C-C motif chemokine ligand 3 like 3 (*CCL3L3*), CD83 molecule (*CD83*), and prostaglandin E receptor 4 (*PTGER4*) were closely related to the processes of leukocyte cell–cell adhesion, mononuclear cell migration, and myeloid leukocyte migration (Figures 6D–F).

### Protein-Protein Interaction Network Analyses of the IRDEGs

**FIGURE 7:**
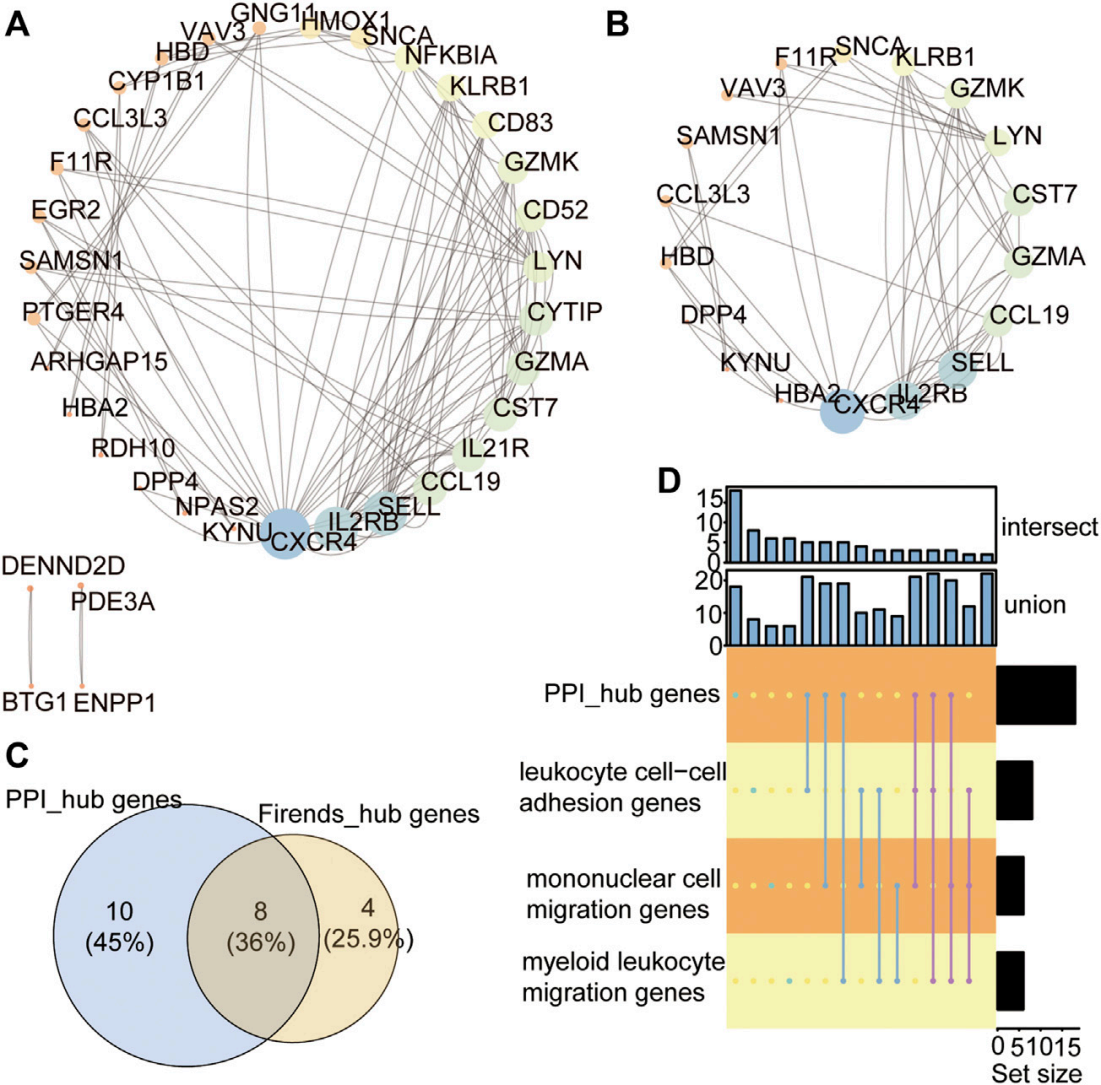
Protein-Protein interaction network analyses of the IRDEGs. **(A)** PPI network showing the protein-protein interaction coded by IRDEGs. **(B)** The PPI_hub_genes with the rank >10. **(C)** Venn showing the 8 hub genes between the PPI_hub_genes and functional similarities_hub_genes. **(D)** The intersection of genes in PPI hub genes and in GO terms of top 3.

Then, the protein–protein interaction (PPI) network of the 68 IRDEGs in ATAAD was constructed to further identify the hub genes of ATAAD (Figure 7A). cytoHubba of Cytoscape software was used to analyse the hub genes, and a total of 18 genes with the highest scores were identified as hub genes (Figure 7B). To distinguish these genes from the previously identified hub genes from the functional similarities analysis, we named these genes PPI_hub genes. These PPI_hub genes were then combined with the previous 12 hub genes found by the functional similarities analysis (functional similarities_hub genes) in Figure 6C via a Venn diagram, and we identified eight hub genes that were closely related to immune infiltration (Figure 7C), including *CXCR4*, LYN proto-oncogene, Src family tyrosine kinase (*LYN*), *CCL19*, *CCL3L3*, *SELL*, F11 receptor (*F11R*), Dipeptidyl peptidase 4 (*DPP4*), and vav guanine nucleotide exchange factor 3 (*VAV3*). Meanwhile, the cogenes between the PPI network and the GO terms of the top 3 are also shown by the UpSet plot (Figure 7D).

### Immune Cell Infiltration Analysis of IDEG Subtypes

**FIGURE 8:**
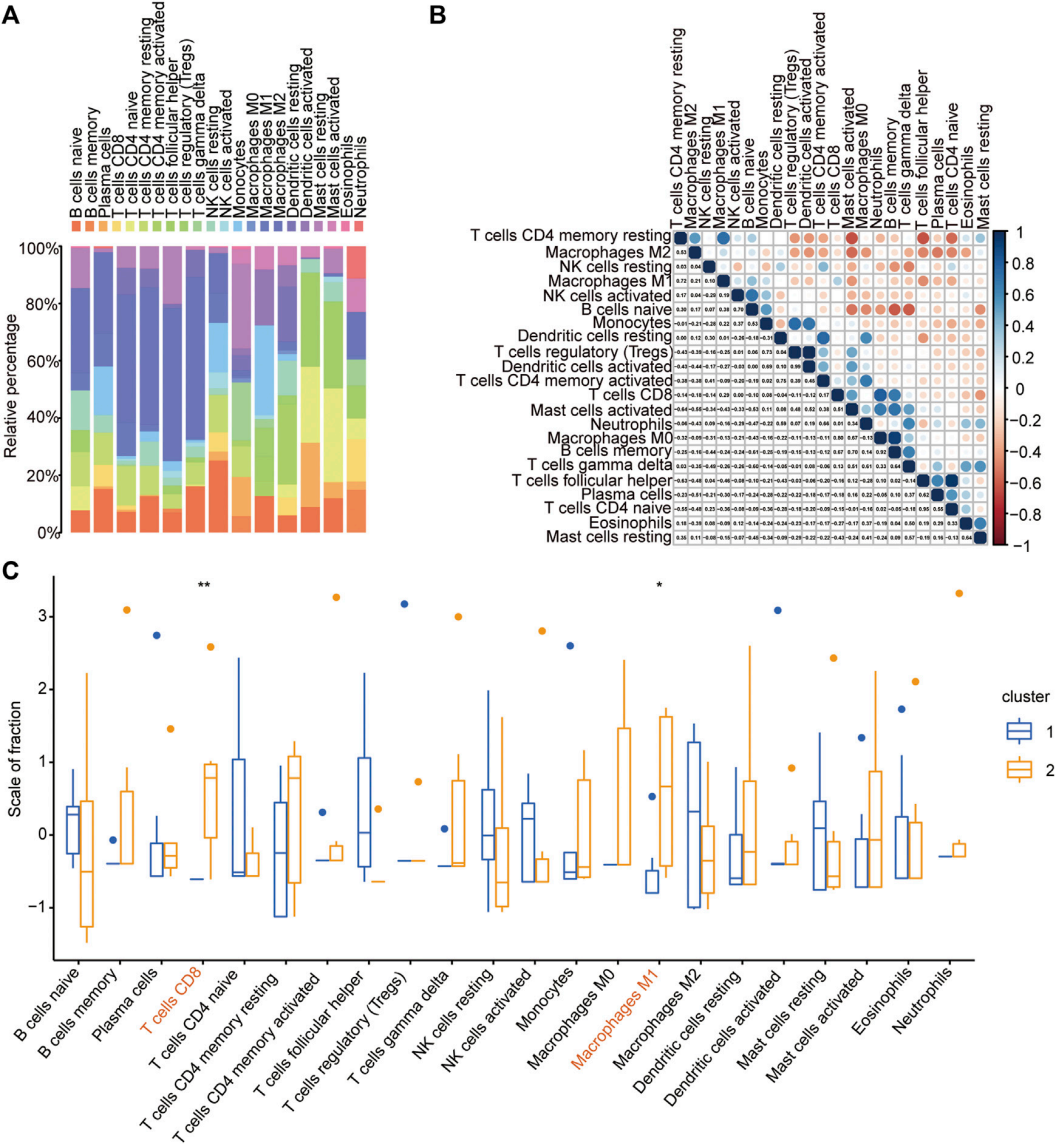
Immune cell infiltration analysis of IDEG subtypes. **(A)** The landscape of the 22 types of immune cells infiltration in ATAAD samples based on CIBERSORT algorithm. **(B)** The correlation of the 22 types of immune cells. **(C)** The comparison of the 22 immune cells infiltration proportion between the two immune-related clusters in ATAAD.

To further compare the immune response of ATAAD patients between the two immune-related clusters, the immune cell infiltration degree of the 22 immune cells was calculated by the CIBERSORT algorithm. The landscape of the immune cell infiltration proportion in ATAAD samples showed that T cells and macrophages accounted for the main types of cells among the 22 types of immune cells (Figure 8A). Correlation analyses of the 22 immune cells showed that resting memory CD4 T cells were positively correlated with M2 macrophages and M1 macrophages (Figure 8B). Activated NK cells were positively correlated with naïve B cells (Figure 8B). Monocytes were positively correlated with regulatory T cells and active dendritic cells (Figure 8B). Resting dendritic cells were positively correlated with activated memory CD4 T cells (Figure 8B). CD8 T cells were positively correlated with neutrophils (Figure 8B). Notably, M0 macrophages showed the strongest positive correlation with memory B cells and T follicular helper cells showed the strongest positive correlation with naïve CD4 T cells (Figure 8B). Moreover, resting memory CD4 T cells were negatively correlated with activated mast cells and follicular helper T cells and naïve B cells were negatively correlated with memory B cells and gamma delta T cells (Figure 8B). The CIBERSORT algorithm results showed that the proportions of CD8 T cells and M1 macrophages were significantly higher in ATAAD patients in cluster 2 than cluster 1 (Figure 8C). These results indicated that the two clusters differentiated by these immune-related genes had distinct immune responses and that the high immune cluster had a better immune response effect.

## Discussion

Aortic dissection (AD) is a life-threating disease that requires timely diagnosis and surgical treatment, with an incidence of 35 cases per 100,000 person each year in those 65–75 years of age (Nienaber et al., 2016). The combined effects of aortic wall stress and aortic wall interlayer abnormalities cause the media rupture and intima tearing, as well as the subsequent penetration of blood, and then splits the aortic wall layers. A so-called false lumen was formed in the mesosphere, that is separated from the inherent true lumen by the dissection membrane (Hiratzka et al., 20102010; Silaschi et al., 2017), leading to destruction of the adventitia (aortic rupture) or a tear in the second anatomic membrane, which allows blood to re-enter the true lumen. ATAAD is the most severe form of AD, that is classified as Stanford-A when the ascending aortic thoracic tract and/or the arch are involved (Cifani et al., 2015; Jiang and Si, 2019). Several risk factors have been identified that can damage the aortic wall and lead to dissection, including poorly controlled hypertension, inherited connective tissue diseases (ITCDs) (Bicuspid aortic valve, Marfan syndrome, Loeys-Dietz syndrome, and Ehler-Danlos syndrome), vascular inflammation, and history of cardiac surgery and previous trauma (Mehta et al., 2002; Januzzi et al., 2004; Goldfinger et al., 2014). Early clinical symptoms of ATAAD may be similar to other disease, including acute coronary syndrome, pulmonary embolism, or pneumothorax, that often leads to delayed diagnosis (Spittell et al., 1993; Clough and Nienaber, 2015; Fukui, 2018). Therefore, early diagnosis and prompt treatment are important for the improvement of survival (Hagan et al., 2000; Nienaber et al., 2016; Lian et al., 2021).

Although an understanding of the pathological mechanism that influences the development of ATAAD has been developed, the molecular pathways underlying this disease have been difficult to identify. Previous studies have reported that the systemic immune-inflammation index is a reliable biomarker for predicting short-term outcomes in ATAAD patients undergoing surgery (Xu et al., 2022), and could be used for ATAAD patient stratification and selection (Samanidis et al., 2022). Inflammation may also be a risk factor leading to weakening of the aortic wall in ATAAD (Del Porto et al., 2014). Arterial wall remodelling depends upon the complex interaction between cells, proinflammatory mediators, and MMPs, which are regulated by an immune response (Cifani et al., 2015). However, the immune infiltration-related gene signature in ATAAD is unclear.

In the present study, we provided evidence that eight innate immune-related genes may serve as potential diagnostic biomarkers or therapeutic targets for ATAAD. Through an analysis of samples obtained from the GEO database, we found that the ATAAD sample could be divided into two notably different subgroups with distinct degrees of immune cell infiltration after the ssGSEA score analyses, WGCNA analyses, and consistent unsupervised cluster analyses. The ssGSEA results confirmed that the two independent subgroups were sufficient to account for the expression differences of immune-related genes in the GEO datasets. Additionally, a total of 68 genes showed significantly different expression between the two immune-related gene subgroups. The subsequent GO, KEGG, GSEA, and GSVA functional enrichment analyses showed that these IRDEGs were enriched in the biological processes of leukocyte cell–cell adhesion, regulation of lipid metabolic process, mononuclear cell migration, negative regulation of cell activation, myeloid leukocyte migration, and regulation of B-cell proliferation and pathways of chemokine signaling pathway, and viral protein interaction with cytokine and cytokine receptor. Leukocyte migration predicts inflammation formation because these cells migrate to the inflammation sites to eliminate the primary inflammatory signals and finally contribute to wound healing and tissue repair (Shi and Pamer, 2011; Nourshargh and Alon, 2014; Cifani et al., 2015).

According to the PPI network and functional similarities analyses, eight of these IRDEGs were identified as hub genes involved in immune regulation in ATAAD. The eight hub genes were all closely related immune genes. *CXCR4* encodes a CXC chemokine receptor specific for stromal cell-derived factor-1, which acts with the CD4 protein to support HIV entry into cells. Among the chemokine receptors, CXCR4 stands out for its pleiotropic roles in several pathological conditions, including immune diseases, viral infections and cancers (Pozzobon et al., 2016). Because of its immune response functions, CXCR4 has been shown to be crucial for the formation of the T-cell immune response as well as the homing, development, and function of B cells (Molon et al., 2005). *LYN* is reported to be an essential regulator of immunoreceptor signalling that initiates both proinflammatory and suppressive signalling pathways in myeloid immune cells, including neutrophils, dendritic cells, monocytes, macrophages, and B lymphocytes (Brian and Freedman, 2021), whose deficiency leads to the development of autoimmune disease (Hibbs and Dunn, 1997). *CCL19* is one of several CC cytokine genes clustered on the p-arm of chromosome 9. CCL19 plays a role in normal lymphocyte recirculation and homing, in T-cell trafficking in the thymus, and in T-cell and B-cell migration to secondary lymphoid organs, which improves immune cell infiltration (Adachi et al., 2018). *CCL3L3* is also considered a proinflammatory M1 in the cancer microenvironment (Zeiner et al., 2019). *SELL* encodes a cell surface adhesion molecule that belongs to a family of adhesion/homing receptors and acts to promote the migration of leukocytes to lymphoid organs (Wedepohl et al., 2012; Xu et al., 2021). *F11R* encodes an important regulator of tight junction assembly in epithelia that can act as a receptor for reovirus, a ligand for the integrin LFA1, or a receptor for platelets. F11R is required for the proliferation and migration of inflamed smooth muscle cells, thereby playing an important role in the subsequent growth of atherosclerotic plaques (Azari et al., 2010). *DPP4* is a transmembrane serine protease that cleaves off N-terminal dipeptides (Chitadze et al., 2021; Love and Liu, 2021), which are highly involved in glucose and insulin metabolism as well as in immune regulation. *VAV3* encodes the protein of a Rho family GTPase that regulates cell signalling pathways, including those of T- and B-cell receptors, by mediating the activities of Rho family members (Shen et al., 2017). These hub genes were all downregulated in the high-immune infiltration subtype compared to the low-immune infiltration subtype, suggesting that these genes play a critical role in immune cell infiltration in ATAAD.

The infiltrating proportion of immune cell subsets detected by CIBERSORT always reflects the time course of innate and adaptive immune responses in human disease (Kawada et al., 2021), which provides novel insights into the pathogenesis of ATAAD. Here, we found that in the high-immune cluster (Cluster 2) of ATAAD, the proportions of CD8 T cells and M1 macrophages were significantly higher than those in the low-immune cluster (Cluster 1). M1 macrophages are typically induced by Th1 cytokines and secrete higher levels of proinflammatory cytokines, such as TNF-α, IL-1α, IL-1β, IL-6, IL-12, and IL-23. Functionally, these M1 macrophages have robust antimicrobial and antitumoral activity, mediate ROS-induced tissue damage, and impair tissue regeneration and wound healing (Shapouri-Moghaddam et al., 2018). Macrophages have been demonstrated to play a fundamental role in triggering and maintaining the intraparietal inflammation underlying ascending thoracic dissection in patients with ITCD and those with no genetic predisposition (del Porto et al., 2010; Cifani et al., 2015; Del Porto et al., 2014; He et al., 2006; Saraff et al., 2003; He et al., 2008). In addition, macrophages are major factors in the pathogenesis of chronic inflammatory and autoimmune diseases (Wynn et al., 2013; Jiang et al., 2014). CD8^+^ T cells play critical roles in innate and adaptive immune defence mechanisms to protect against extrinsic and intrinsic dangers (Zhang and Bevan, 2011; Schäfer and Zernecke, 2020). Innate immunity is mainly related to the destructive pattern underlying aortic wall rupture (Cifani et al., 2015). The two immune gene-related subgroups had different proportions of infiltrating CD8 T cells and M1 macrophages, indicating a possible difference in the pathological vascular wall in patients with ATAAD.

Our study innovatively performed unsupervised clustering analyses based on the immunoregulatory genes that were screened through ssGSEA and WGCNA, thereby providing a novel theoretical basis for the immune regulation mechanism involved in the progression of ATAAD and promoting a better understanding of the correlation between the development of ATAAD and immune regulation disorder. This study proposes for the first time the linkage between eight immune-related signatures and ATAAD progression. However, certain limitations were observed in the present study. First, the small sample size may lead to some bias in the research results. Thus, additional data should be collected for further analysis. Second, certain postoperative indicators were lacking in this study due to the lack of clinical information in the GEO database. Hence, clinical samples of ATAAD will be collected for high-throughput sequencing, and the corresponding clinical indices would be included, including genetic history, cardiovascular history, and follow-up information after surgery, to further analyse the contribution of immune cell infiltration to ATAAD. Finally, this study is only a bioinformatic analysis based on existing databases, but lacks validation based on experiments. Therefore, animal models would be constructed to verify the potential impact of immune dysregulation on the disease progression. What’s more, cell experiments should also be performed to analyse the molecular mechanism of these eight genes involved in ATAAD.

In summary, our findings reveal the critical role of the innate immune response in the development and progression of ATAAD and suggest that the eight related hub genes identified here may serve as biomarkers and targets for the diagnosis and immunotherapy of patients with ATAAD. In the future, additional attention should be focused on the effect of immune dysregulation on the pathomechanism of ATAAD.

## Data Availability

The original contributions presented in the study are included in the article/Supplementary Material, further inquiries can be directed to the corresponding author.
